# Comparative analysis of catfish BAC end sequences with the zebrafish genome

**DOI:** 10.1186/1471-2164-10-592

**Published:** 2009-12-10

**Authors:** Hong Liu, Yanliang Jiang, Shaolin Wang, Parichart Ninwichian, Benjaporn Somridhivej, Peng Xu, Jason Abernathy, Huseyin Kucuktas, Zhanjiang Liu

**Affiliations:** 1 The Fish Molecular Genetics and Biotechnology Laboratory, Department of Fisheries and Allied Aquacultures and Program of Cell and Molecular Biosciences, Aquatic Genomics Unit, Auburn University, Auburn, AL 36849, USA; 2 College of Fisheries, Huazhong Agricultural University, Wuhan, 430070, China; 3 Key Laboratory of Protein Chemistry and Developmental Biology of State Education Ministry of China, College of Life Sciences, Hunan Normal University, Changsha, 410081, China

## Abstract

**Background:**

Comparative mapping is a powerful tool to transfer genomic information from sequenced genomes to closely related species for which whole genome sequence data are not yet available. However, such an approach is still very limited in catfish, the most important aquaculture species in the United States. This project was initiated to generate additional BAC end sequences and demonstrate their applications in comparative mapping in catfish.

**Results:**

We reported the generation of 43,000 BAC end sequences and their applications for comparative genome analysis in catfish. Using these and the additional 20,000 existing BAC end sequences as a resource along with linkage mapping and existing physical map, conserved syntenic regions were identified between the catfish and zebrafish genomes. A total of 10,943 catfish BAC end sequences (17.3%) had significant BLAST hits to the zebrafish genome (cutoff value ≤ e^-5^), of which 3,221 were unique gene hits, providing a platform for comparative mapping based on locations of these genes in catfish and zebrafish. Genetic linkage mapping of microsatellites associated with contigs allowed identification of large conserved genomic segments and construction of super scaffolds.

**Conclusion:**

BAC end sequences and their associated polymorphic markers are great resources for comparative genome analysis in catfish. Highly conserved chromosomal regions were identified to exist between catfish and zebrafish. However, it appears that the level of conservation at local genomic regions are high while a high level of chromosomal shuffling and rearrangements exist between catfish and zebrafish genomes. Orthologous regions established through comparative analysis should facilitate both structural and functional genome analysis in catfish.

## Background

Comparative mapping is a powerful tool to transfer genomic information from sequenced genomes to closely related species for which whole genome sequence data are not yet available. Such an approach was initially demonstrated by Fujiyama *et al*. [1] for the construction of the human-chimpanzee comparative map. In these closely related primate species, approximately 98% of chimpanzee BAC end sequences (BES) had significant BLAST hits to the human genome sequence allowing putative orthologues to be identified [1]. A similar approach was used for the construction of the human-mouse comparative map [2]. Subsequently, this approach was extensively used in mammals including construction of the human-cattle, the human-horse, and the human-porcine comparative maps [3-5]. Most recently, this approach was utilized one step further for the construction of the comparative genome contig (CGC)-based physical map of the sheep genome [6], where CGC is established based on anchorage of the sheep BES onto the genome sequences of dog, cow, and human. These successes depended on high percentage of BLAST hits and/or high levels of genome collinearity.

Five teleost fish genomes have been fully sequenced http://www.ensembl.org/index.html including zebrafish (*Danio rerio*, from the order of Cypriniformes), Japanese pufferfish (*Fugu rubripes*, from the order of Tetraodontiformes), green spotted pufferfish (*Tetraodon nigroviridis*, from the order of Tetraodontiformes), medaka (*Oryzias latipes*, from the order of Beloniformes), and three-spined stickleback (*Gasterosteus aculeatus*, from the order of Gasterosteiformes), while whole genome sequencing is also underway for tilapia http://www.cichidgenome.org; http://www.broad.mit.edu/science/projects/mammals-models/vertebrates-invertebrates/tilapia/tilapia-genome-sequencing-project. The availability of these whole genome sequences lends great opportunities for comparative genome analysis. Recently, major genomic resources have been developed from a number of fish species such as Atlantic salmon (*Salmo salar*) [7-9], rainbow trout (*Oncorhynchus mykiss*) [10,11], tilapia [12,13], gilthead sea bream (*Sparus auratus*) [14-17], European sea bass (*Dicentrarchus labrax*) [18,19], and channel catfish (*Ictalurus punctatus*) (for a review, see [20,21]).

Catfish is the major aquaculture species in the United States. It is one of the six species included in the U.S. National Animal Genome Project NRSP-8. A number of genome resources have been developed in catfish including a large number of molecular markers [22-25], genetic linkage maps [26-28], several hundred thousands of ESTs ([29-33]; Z. Liu, unpublished data), microarray platforms [34-38], BAC libraries [39,40], and BAC-based physical maps [41,42]. To enable BAC end sequence-based comparative genome analysis, we previously reported generation of 20,366 BES in catfish [25]. In spite of the great value of those BES for the characterization of genome repeat structures [43] and for the identification of microsatellite markers, our previous comparative genome analysis using BES revealed very limited conservation between the catfish and zebrafish genomes. Of the 141 mate-paired BES with genes on both ends of the BAC inserts, only 34 (24.1%) were found in nearby genomic locations in the zebrafish genome, suggesting high levels of chromosomal rearrangements [40]. Such findings were in strong contrast to the situations found between medaka-sea bream, *Tetraodon*-sea bream, medaka-stickleback, *Tetraodon*-medaka, stickleback-sea bream, *Tetraodon*-stickleback genome comparisons where almost complete genome collinearities were found [44]. We speculated that our earlier inability to discover large extent of genome collinearity between catfish and zebrafish could be a result of the low numbers of BES and the lack of a physical map. Therefore, in this study, we extended our efforts in BAC end sequencing and generated additional 43,021 BES, bringing the total to 63,387 (25,676 mate-paired). Using these catfish BES and its BAC contig-based physical map [42], genetic linkage mapping of BAC end-anchored microsatellites, and the genome sequence of zebrafish, here we conducted extensive comparative genome analysis. We report the identification of conserved syntenies and demonstrate the construction of super scaffolds of contigs by genetic linkage mapping of BAC end-associated microsatellites.

## Results and Discussion

### BAC end sequencing

As shown in Table 1, a total of 42,240 BAC inserts (6.13× clone-coverage of the channel catfish genome) were sequenced from both ends, resulting in 63,387 BES ≥ 200 bp in length (75% overall success rate), including 20,366 BES we previously reported [25]. Mate-paired BES were produced from 25,676 BAC clones, while only a single BES was obtained from 12,035 clones. The BES were of high quality as the Q20 length ranged from 200 to 810 bp, with an average Q20 read length of 596 bp. All these BES have been deposited into the GenBank GSS database with consecutive accession numbers of [GenBank:FI857756-FI900776]. A total of 37,784,877 bp of genomic sequences was generated from this study, representing approximately 4% of the catfish genome. Analysis using the 37,784,877 bp BES resulted in 11.91% of base pairs masked using the *Danio *repeat database, with the most abundant type of repeat being the DNA transposons. We previously reported the assessment of repetitive elements in the catfish genome and the additional 43,021 BES generated in this study confirmed our previous findings in general [25]. These BES [GenBank:DX083364-DX103729] were also used for comparative genome analysis in this study.

**Table 1:** A summary of BAC end sequences

Category	Numbers
BAC sequence reactions	84,480
Total clean sequences	63,387 (75% success)
T7 sequences	32,074
SP6 sequences	31,313
Pair BAC end sequences	25,676
Total length sequenced	37,784,877 bp
Average length	596 bp

### *In silico *analysis of the BAC-associated catfish genes on the zebrafish genome

TBLASTX searches using the 63,387 catfish BES against the ENSEMBL zebrafish cDNA database with chromosome information resulted in 5,066 significant hits (Table 2). Of the 5,066 significant hits, 2,197 unique zebrafish genes were hit by a single BES while 1,024 unique zebrafish genes were hit by two or more catfish BES, making a total of 3,221 unique zebrafish genes with significant hits from the catfish BES. The 3,221 genes cover all 25 zebrafish chromosomes, with the largest number of gene hits being located on chromosome 5 (224 significant hits), followed by chromosome 7 (191 significant hits), chromosome 20 (171 significant hits), chromosome 6 (151 significant hits) and chromosome 19 (134 significant hits); and the smallest number of gene hits on chromosome 24 with 78 hits (Table 2). The number of gene hits on various chromosomes was approximately proportional to the sizes of the zebrafish chromosomes with some exceptions. When the size of chromosomes was taken into consideration, chromosome 25 had the largest number of gene hits with 3.5 hits per Mb or one hit per 286 kb on average, followed by chromosome 5, 4, 20, 19, and 22 with 3.2, 3.1, 3.0, 2.9, and 2.9 hits per Mb, respectively (Table 2).

**Table 2:** Distribution of comparatively anchored BAC clones using protein encoding gene sequences only.

Zebrafish chromosome	Chromosome size (Mb)	No. of protein encoding genes*	No. of tBLASTx hits	Hits to unique genes	Unique gene hits per Mb	No. of contigs with single gene hits	No. of contigs with multiple gene hits	No. putative micro-syntenies
1	56.2	818	205	123	1.83	75	17	13
2	54.4	875	194	133	2.13	85	15	13
3	62.9	975	196	127	1.75	72	18	13
4	42.6	743	221	130	2.77	78	16	9
5	70.4	1,173	340	224	2.74	103	35	21
6	59.2	818	232	151	2.31	85	22	18
7	70.3	990	283	191	2.33	80	34	25
8	56.5	864	196	128	1.93	65	22	14
9	51.5	700	212	133	2.17	61	21	16
10	42.4	670	150	87	1.72	51	12	9
11	44.6	627	161	121	2.26	67	16	10
12	47.5	636	177	114	2.21	72	14	7
13	53.5	744	200	113	1.96	67	21	14
14	56.5	701	197	113	1.77	84	11	7
15	46.6	688	177	125	2.25	68	14	8
16	53.1	773	181	124	2.02	77	14	10
17	52.3	715	180	115	1.99	62	19	13
18	49.3	749	193	121	2.23	47	22	21
19	46.2	780	233	134	2.58	86	21	16
20	56.5	1,053	277	171	2.48	76	30	19
21	46.1	721	163	117	2.28	63	14	10
22	39.0	959	178	113	2.59	50	19	16
23	46.4	669	204	121	2.24	68	18	13
24	40.3	513	117	78	1.71	47	8	7
25	32.9	597	199	114	3.04	65	19	14

**Total/Average**	**1,277.2**	**19,551**	**5,066**	**3,221**	**2.21**	**1,754**	**472**	**336**

*: Annotated genes only from ENSEMBL.

One particular finding of these BLAST searches is the observation of many highly repetitive genes. Out of 3,221 unique genes, 1,024 genes had hits from two or more BES. A single gene identity had hits from as many as 31 BES. A total of 14 genes had hits from at least 10 BES each (Table 3); an additional 139 genes had hits from 4-9 BES each; 230 genes had hits from 3 BES each, and 641 genes had hits from 2 BES each (Table 3). Some of the genes with hits from multiple BES may represent a whole array of related genes with similar functional domains. For instance, 18 BES hit NOD3-like gene of channel catfish, which was just recently characterized; NOD3 gene existed as a single copy gene in the catfish genome [45], and apparently the multiple BES contained many related genes harboring domains present within the NOD3 gene. Theoretically, a fraction of genes should have hits by more than one BES, simply because of the genome coverage of the BAC clones. We believe that overlapping (including identical) BAC clones does account for some of the observed hits of genes by more than one BES (data not shown), especially for those with 2-3 BES hits. However, the mathematical chances do not support multiple BES hits of a single gene unless the gene itself is repetitive in the catfish genome. Additional research is warranted to fully understand the nature of these genes/sequences in the catfish genome, but clearly many of these represent classes of repetitive gene families such as DNA polymerase gene that had hits from 31 BES.

**Table 3:** Distribution of genes with hits from multiple BAC end sequences, with details provided for genes with 10 or more hits from BAC end sequences

*No. of Genes*	Putative Identities	*No of BES hits *	Presence in Zebrafish genome	Potential explanation
1	Novel protein similar to DNA polymerases	31	28	Repetitive elements related to retroelements
1	Methionine aminopeptidase 1	22	2	Repetitive elements or multigene family
1	NOD3 protein-like	18	63	Common domains shared by many related proteins
1	Similar to tudor domain containing 7, hypothetical protein LOC393661	17	89	Repetitive elements or repetitive genes
1	Similar to porf2	16	81	Repetitive elements or multigene family
1	Similar to general transcription factor II-I repeat domain-containing protein 2A	16	82	Repetitive elements or multigene family
1	Similar to novel G protein-coupled receptor	13	92	Repetitive elements or multigene family
1	Similar to serine/threonine-protein kinase pim-3;	11	85	Repetitive elements or multigene family
1	Similar to novel protein from *Danio rerio*;	11	85	Repetitive elements or multigene family
1	Similar to Dynein heavy chain 6	11	20	Repetitive elements or multigene family
1	ORF2 [*Mus musculus domesticus*]	10	91	Repetitive elements or multigene family
1	PREDICTED: tubulin, alpha, ubiquitous isoform 8 [*Macaca mulatta*]	10	16	Repetitive elements or multigene family
1	PREDICTED: similar to vacuolar protein sorting 52 [*Danio rerio*]	10	69	Repetitive elements or multigene family
1	GF20795 [*Drosophila ananassae*]	10	4	Repetitive elements or multigene family
**14**		**Subtotal **		
68		5-9		Repetitive elements or multigene family
71		4		Repetitive elements or multigene family
**139**		**Subtotal**		
230		3		Potentially duplicated gene candidates
641		2		Potentially duplicated gene candidates
**1024**		**Total**		

### Establishing microsyntenies

Among the teleost genomes with high sequence coverage, zebrafish is the most closely related species to catfish [46]. Our initial BLAST searches of the catfish BES against the genome of the *T. nigroviridis *generated many fewer significant hits compared to those against the zebrafish genome. Therefore, we concentrated our comparative analysis efforts with the zebrafish genome in this study.

Conserved syntenies are most often established by comparing genome sequences of related species. However, the whole genome sequence is not yet available from catfish. In the absence of the whole genome sequence, we attempted to establish microsyntenies based on physical linkage of gene sequences. With the genome resources available in catfish, we have taken three approaches. First, if the genes were identified from both ends of a single BAC clone, they are physically linked with a distance of the BAC clone insert size. If the same two genes are found linked in the zebrafish genome in the same genome neighborhood, a microsynteny can then be established. These genes from mate-paired BES are physically linked with the average distances between them being the average insert size of the catfish BAC library, i.e., 161 kb. From the 63,387 BES, a total of 25,676 mate-paired BES were identified. Of these, 760 mate-paired BES had significant BLASTN hits against the zebrafish genome sequence. However, only 194 of the 760 significant hit pairs were on the same zebrafish chromosome, allowing syntenic comparison. Further tBLASTX searches against the ENSEMBL zebrafish cDNA database allowed identification of 95 mate-paired BES with genes on both sides. The genomic locations of these 95 mate-paired genes were determined from the zebrafish genome sequence. Fifty pairs were found to be present in neighboring genomic locations within one million base pairs, while the other 45 were present in more distant locations (> 1 Mb) on the same chromosomes. The vast majority of the 50 mate-paired genes were found to be within 500 kb on the zebrafish genome sequence; only 2 of the 50 pairs had a distance of 500-920 kb (Table 4), suggesting conserved syntenies of the involved genes.

**Table 4:** Summary of 50 conserved syntenies identified by comparison of 95 mate-paired genes of channel catfish with genomic locations of those on the zebrafish draft genome sequence.

Catfish BAC ID	SP6 hits	T7 hits	zebrafish Chr	Chr location (Mb)	Distance (bp)
035I16	[GenBank:NP_001038735]	[GenBank:XP_689146]	2	38.61	18,048
063L16	[GenBank:NP_001012377]	[GenBank:NP_001092217]	2	19.25	48,679
026B05	[GenBank:XP_001921249]	[GenBank:CAI20867]	3	31.82	4,418
098D05	[GenBank:CAI11873]	[GenBank:NP_001038462]	4	1.82	301,470
007M06	[GenBank:XP_001333162]	[GenBank:NP_571105]	5	5.46	379,579
028M04	[GenBank:XP_687685]	[GenBank:NP_958867]	5	22.23	133,063
074L07	[GenBank:XP_001334912]	[GenBank:XP_687570]	5	15.69	290,598
035N11	[GenBank:NP_001032187]	[GenBank:NP_958882]	6	18.25	231,524
040J24	[GenBank:XP_686613]	[GenBank:NP_991309]	6	7.37	443,524
077F04	[GenBank:NP_001034906]	[GenBank:NP_001038813]	6	11.42	484,877
032F20	[GenBank:XP_691291]	[GenBank:NP_001075159]	7	28.40	244,425
062B16	[GenBank:NP_001008651]	[GenBank:NP_001017550]	7	15.05	222,416
075K10	[GenBank:NP_956505]	[GenBank:NP_001070187]	7	13.34	268,455
103I21	[GenBank:NP_998033]	[GenBank:NP_001159825]	7	22.34	293,845
047N13	[GenBank:XP_699919]	[GenBank:XP_699627]	8	23.84	203,260
057N22	[GenBank:XP_001923800]	[GenBank:NP_997066]	9	36.69	163,677
076H22	[GenBank:NP_001004563]	[GenBank:XP_001921910]	9	13.84	313,352
105N24	[GenBank:NP_001122018]	[GenBank:NP_997992.2]	9	27.37	268,271
056A23	[GenBank:NP_998295]	[GenBank:NP_001103164]	10	7.50	486,745
068C21	[GenBank:NP_001019272]	[GenBank:XP_001920077]	10	17.72	104,067
093K02	[GenBank:XP_001344325]	[GenBank:XP_001919728]	10	41.67	152,479
096A14	[GenBank:NP_001120805]	[GenBank:NP_997775]	11	10.02	980,276
010B15	[GenBank:NP_956715]	[GenBank:NP_956756]	12	25.17	111,625
041B24	[GenBank:NP_001002607]	[GenBank:XP_693784]	12	13.59	398,886
109K22	[GenBank:XP_001920588]	[GenBank:XP_001920550]	12	25.55	449,096
018H11	[GenBank:NP_956915]	[GenBank:NP_997743]	13	23.23	293,181
026C08	[GenBank:NP_001119869]	[GenBank:NP_956611]	13	23.50	201,663
027E09	[GenBank:XP_001922173]	[GenBank:XP_001921791]	14	0.22	574,761
103F19	[GenBank:NP_571477]	[GenBank:XP_001919973]	14	22.90	256,143
022D09	[GenBank:NP_001096112]	[GenBank:XP_684421]	15	29.14	418,219
059B20	[GenBank:XP_682817]	[GenBank:XP_691794]	15	27.80	94,396
077H08	[GenBank:XP_001920240]	[GenBank:NP_001070609]	15	6.05	339,215
004O03	[GenBank:NP_001020707]	[GenBank:NP_001020642]	16	10.40	149,618
075M09	[GenBank:NP_001107266]	[GenBank:NP_001082899]	16	8.30	120,355
104I08	[GenBank:NP_571871]	[GenBank:NP_694503]	16	27.17	214,517
013P16	[GenBank:NP_001076304]	[GenBank:NP_001038173]	18	18.65	366,849
042J20	[GenBank:NP_001037796]	[GenBank:NP_001038370]	18	32.85	223,781
021L13	[GenBank:NP_001038343]	[GenBank:XP_688911]	19	17.40	286,019
052N18	[GenBank:XP_001921158]	[GenBank:NP_956134]	19	7.59	345,553
056A09	[GenBank:NP_001018463]	[GenBank:NP_001038416]	19	13.46	122,217
065M02	[GenBank:XP_001922809]	[GenBank:NP_001038686]	19	15.37	430,325
080O21	[GenBank:NP_956277]	[GenBank:NP_571262]	19	17.06	389,643
086C16	[GenBank:XP_001340912]	[GenBank:NP_001038562]	19	6.00	364,590
010H22	[GenBank:NP_998602]	[GenBank:XP_001920851]	21	2.24	240,019
034I14	[GenBank:NP_001038838]	[GenBank:NP_956334]	21	20.16	306,102
081J10	[GenBank:NP_001002411]	[GenBank:NP_001073439]	21	22.12	277,818
099H22	[GenBank:NP_001076277]	[GenBank:XP_699221]	22	7.03	115,754
053P11	[GenBank:NP_001002173]	[GenBank:NP_001035137]	23	30.73	197,703
068O10	[GenBank:XP_001339131]	[GenBank:XP_689922]	23	18.01	332,973
105H10	[GenBank:NP_001103636]	[GenBank:NP_001098596]	23	33.36	274,318
**Average**					**278,648**

We previously reported the relatively high levels of local region conservation. For instance, many genes within the bordering mate-paired genes were well conserved among catfish, zebrafish, and *Tetraodon*, as determined by direct sequencing of the catfish BAC DNA using primers predicted from known genes in zebrafish or Tetraodon [40]. We did not extend this part of the study, but all known genomic information suggested high levels of local genome conservation.

**Figure 1:**
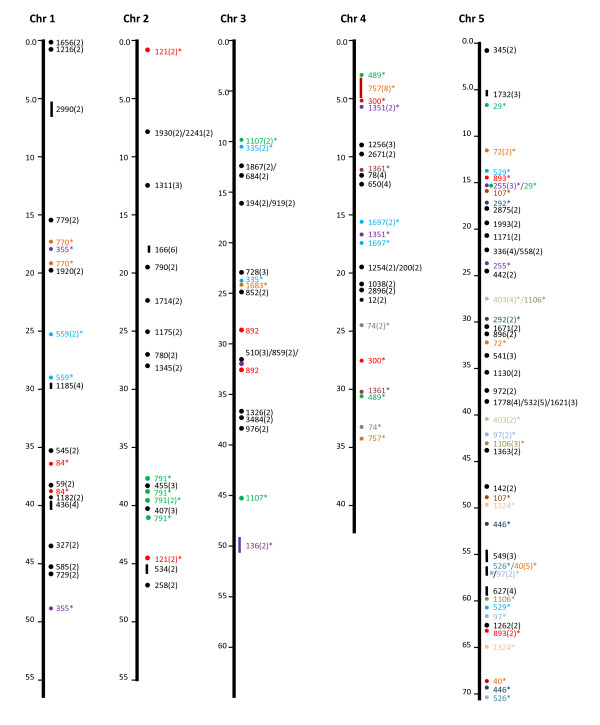
**Identification of microsyntenies through comparative sequence analysis (chr 1 through chr 5)**. tBLASTX searches were conducted using BAC contig-associated BAC end sequences as queries against the zebrafish genome sequence. The putative conserved microsyntenies are presented along the 25 zebrafish chromosomes (chr 1 through chr 25). The position of the zebrafish sequence is shown on the left of each chromosome bars in million base pairs. The conserved microsyntenies are indicated on the right side of the chromosome bars, with the numbers representing the contig numbers of the BAC assembly of the catfish physical map [41]. Circles represent short syntenic regions and short vertical lines represent relatively longer conserved syntenic regions proportional to the length of the bar with a number in parenthesis representing the number of conserved sequences within the microsyntenies. The microsyntenies designated with asterisks (*) are those with duplicated conservation of the microsyntenies that are color-coded to facilitate the visualization of the duplicated syntenic regions along the chromosome. Duplicated syntenic regions refer to a conserved genomic segment between the catfish genome and the zebrafish genome that is duplicated in the zebrafish genome such that identical or nearly identical significant hits are generated from two chromosomal regions of the zebrafish genome using a single catfish genome segment (say it is a contig or a scaffold) as the query. In just few cases, this term is used in an extended fashion to include those that are tripled in the zebrafish genome.

**Figure 2:**
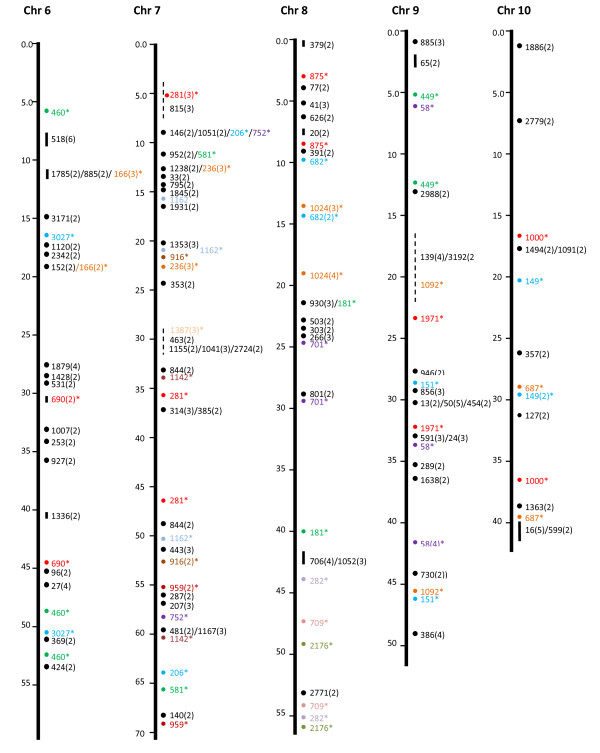
**Identification of microsyntenies through comparative sequence analysis (chr 6 through chr 10)**. tBLASTX searches were conducted using BAC contig-associated BAC end sequences as queries against the zebrafish genome sequence. The putative conserved microsyntenies are presented along the 25 zebrafish chromosomes (chr 1 through chr 25). The position of the zebrafish sequence is shown on the left of each chromosome bars in million base pairs. The conserved microsyntenies are indicated on the right side of the chromosome bars, with the numbers representing the contig numbers of the BAC assembly of the catfish physical map [41]. Circles represent short syntenic regions and short vertical lines represent relatively longer conserved syntenic regions proportional to the length of the bar with a number in parenthesis representing the number of conserved sequences within the microsyntenies. The microsyntenies designated with asterisks (*) are those with duplicated conservation of the microsyntenies that are color-coded to facilitate the visualization of the duplicated syntenic regions along the chromosome. Duplicated syntenic regions refer to a conserved genomic segment between the catfish genome and the zebrafish genome that is duplicated in the zebrafish genome such that identical or nearly identical significant hits are generated from two chromosomal regions of the zebrafish genome using a single catfish genome segment (say it is a contig or a scaffold) as the query. In just few cases, this term is used in an extended fashion to include those that are tripled in the zebrafish genome.

**Figure 3:**
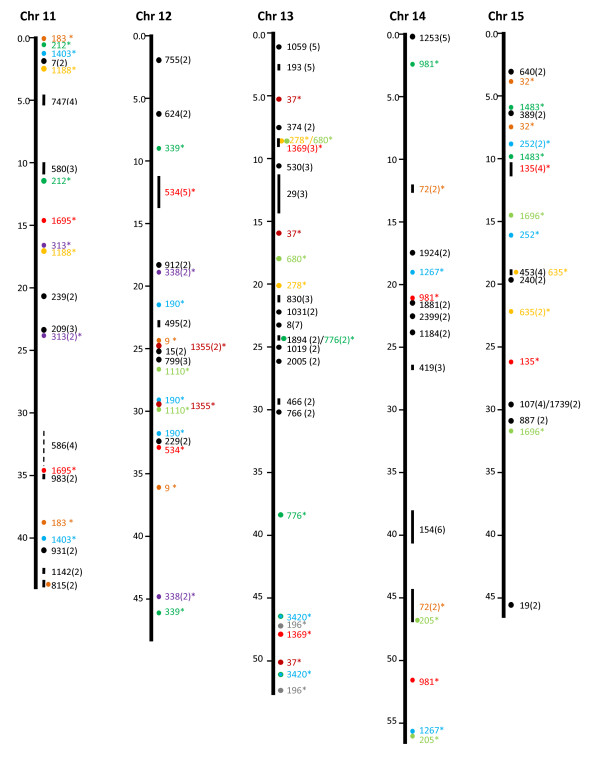
**Identification of microsyntenies through comparative sequence analysis (chr 11 through chr 15)**. tBLASTX searches were conducted using BAC contig-associated BAC end sequences as queries against the zebrafish genome sequence. The putative conserved microsyntenies are presented along the 25 zebrafish chromosomes (chr 1 through chr 25). The position of the zebrafish sequence is shown on the left of each chromosome bars in million base pairs. The conserved microsyntenies are indicated on the right side of the chromosome bars, with the numbers representing the contig numbers of the BAC assembly of the catfish physical map [41]. Circles represent short syntenic regions and short vertical lines represent relatively longer conserved syntenic regions proportional to the length of the bar with a number in parenthesis representing the number of conserved sequences within the microsyntenies. The microsyntenies designated with asterisks (*) are those with duplicated conservation of the microsyntenies that are color-coded to facilitate the visualization of the duplicated syntenic regions along the chromosome. Duplicated syntenic regions refer to a conserved genomic segment between the catfish genome and the zebrafish genome that is duplicated in the zebrafish genome such that identical or nearly identical significant hits are generated from two chromosomal regions of the zebrafish genome using a single catfish genome segment (say it is a contig or a scaffold) as the query. In just few cases, this term is used in an extended fashion to include those that are tripled in the zebrafish genome.

In addition to the 50 microsyntenies, we attempted to determine if significant gene hits in the same catfish BAC contigs also fall on the same chromosome locations comparable to the contig sizes. As shown in Table 2, of the contigs with gene hits, 1,754 contigs had only one gene hit, while 472 contigs had two or more gene hits within each contig. Because the genes in the same contig are physically linked, their linkage in a comparable distance in the zebrafish genome would indicate a conserved synteny. As shown in Figure 1, 2, 3, 4 &5, the vast majority of gene hits within the same contigs were found to be located on the same zebrafish chromosomes with comparable distances as estimated from the catfish BAC contigs. Using such an approach, a total of 336 conserved microsyntenies was identified (Table 2). Presence of multiple gene hits within large BAC contigs would allow identification of extended large conserved syntenic regions. Many of the microsyntenies were conserved with extended genomic distance to span over several million base pairs (Figure 1, 2, 3, 4 &5, for additional details, see Additional file 1). For instance, large conserved syntenies were identified from chromosomes 12, 13, 14, 22, 23, 24, and 25 (Figure 1, 2, 3, 4 &5). In spite of the identification of some relatively large conserved syntenic regions, the vast majority of the identified syntenies were microsyntenies. Such highly segmented microsyntenies are not very useful for genome-wide comparative analysis. However, if scaffolds can be established by determining the relationships among the microsyntenies, large-scale genome comparison should be possible. We, therefore, used two zebrafish chromosomes as the query to demonstrate if super scaffolds can be established. Chromosome 7, one of the chromosomes with the highest number of significant gene hits, and chromosome 13, one of the chromosomes with a large number of contigs having two or more hits (indicative of high level of syntenic conservation), were chosen for further analysis using genetic linkage mapping.

**Figure 4:**
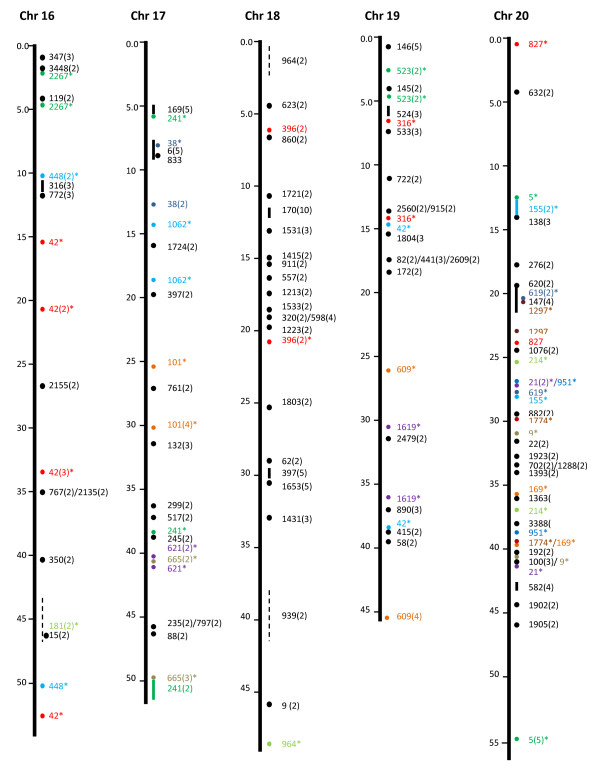
**Identification of microsyntenies through comparative sequence analysis (chr 16 through chr 20)**. tBLASTX searches were conducted using BAC contig-associated BAC end sequences as queries against the zebrafish genome sequence. The putative conserved microsyntenies are presented along the 25 zebrafish chromosomes (chr 1 through chr 25). The position of the zebrafish sequence is shown on the left of each chromosome bars in million base pairs. The conserved microsyntenies are indicated on the right side of the chromosome bars, with the numbers representing the contig numbers of the BAC assembly of the catfish physical map [41]. Circles represent short syntenic regions and short vertical lines represent relatively longer conserved syntenic regions proportional to the length of the bar with a number in parenthesis representing the number of conserved sequences within the microsyntenies. The microsyntenies designated with asterisks (*) are those with duplicated conservation of the microsyntenies that are color-coded to facilitate the visualization of the duplicated syntenic regions along the chromosome. Duplicated syntenic regions refer to a conserved genomic segment between the catfish genome and the zebrafish genome that is duplicated in the zebrafish genome such that identical or nearly identical significant hits are generated from two chromosomal regions of the zebrafish genome using a single catfish genome segment (say it is a contig or a scaffold) as the query. In just few cases, this term is used in an extended fashion to include those that are tripled in the zebrafish genome.

**Figure 5:**
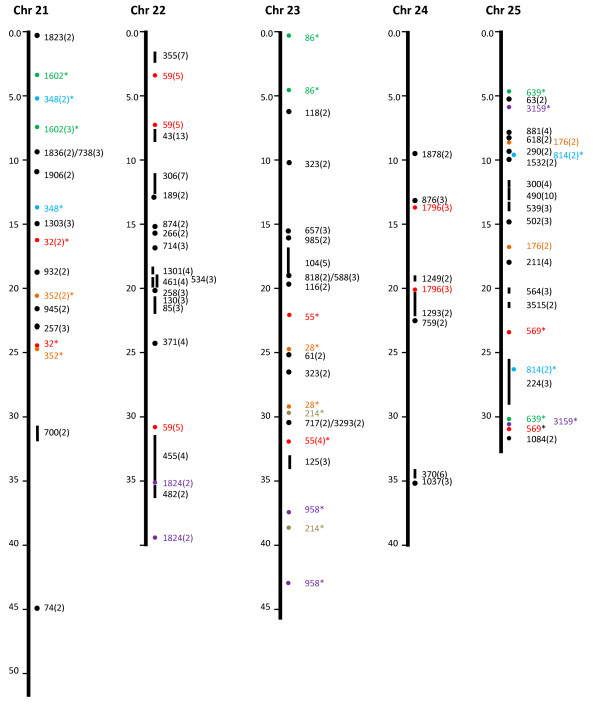
**Identification of microsyntenies through comparative sequence analysis (chr 21 through chr 25)**. tBLASTX searches were conducted using BAC contig-associated BAC end sequences as queries against the zebrafish genome sequence. The putative conserved microsyntenies are presented along the 25 zebrafish chromosomes (chr 1 through chr 25). The position of the zebrafish sequence is shown on the left of each chromosome bars in million base pairs. The conserved microsyntenies are indicated on the right side of the chromosome bars, with the numbers representing the contig numbers of the BAC assembly of the catfish physical map [41]. Circles represent short syntenic regions and short vertical lines represent relatively longer conserved syntenic regions proportional to the length of the bar with a number in parenthesis representing the number of conserved sequences within the microsyntenies. The microsyntenies designated with asterisks (*) are those with duplicated conservation of the microsyntenies that are color-coded to facilitate the visualization of the duplicated syntenic regions along the chromosome. Duplicated syntenic regions refer to a conserved genomic segment between the catfish genome and the zebrafish genome that is duplicated in the zebrafish genome such that identical or nearly identical significant hits are generated from two chromosomal regions of the zebrafish genome using a single catfish genome segment (say it is a contig or a scaffold) as the query. In just few cases, this term is used in an extended fashion to include those that are tripled in the zebrafish genome.

### Genetic mapping of BAC end-anchored microsatellites

In order to extend the scope of conserved microsyntenies, microsyntenies identified on zebrafish chromosomes 7 and 13 were genetically mapped to determine their chromosomal locations in the catfish genome. There were 373 significant BLASTN hits to zebrafish chromosome 13 involving 178 unique catfish BAC contigs; and 505 significant hits to zebrafish chromosome 7 involving 314 unique catfish BAC contigs. We, therefore, first identified microsatellites from these involved catfish BAC contigs, and then mapped them to the linkage groups when the microsatellites were polymorphic in the resource family. A total of 548 pairs of microsatellite primers were tested, of which 296 from 188 contigs (the details of the polymorphic markers are shown in the Additional file 2) were polymorphic in the resource family. Further analysis using JoinMap 4.0 allowed mapping of 290 microsatellite markers, of which 161 microsatellites were from BES with significant similarity to zebrafish chromosome 7, and 129 microsatellites were from BES with significant similarity to zebrafish chromosome 13.

**Figure 6:**
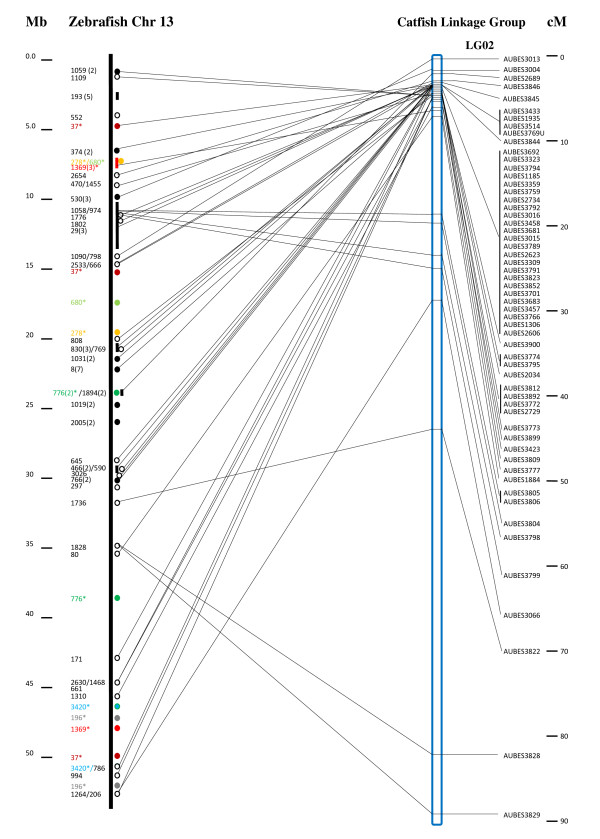
**Scaffolds of conserved syntenic regions between the catfish and zebrafish genomes**. Scaffolds of conserved syntenic regions were established by genetic linkage mapping of BAC contig-associated microsatellites. The zebrafish chromosome 13 (chr 13) is presented with its base positions on the far left in million base pairs; The second column of the numbers are catfish BAC contig numbers [41], with the identified syntenic regions shown immediately right of the chromosome bar. The numbers in the parenthesis are the number of conserved sequences; the circles and bars represent relatively short and long conserved syntenic regions; the asterisks represent duplicated syntenic regions with color coding to facilitate the visualization of duplicated regions, the same way as described under Figure 1 legend, except that the open circles represent conserved sequences coming from non-gene sequences while the solid circles represent conserved gene sequences. Microsatellites from the BAC contigs were genetically mapped to linkage groups as shown on the right, with the names of microsatellites being labeled on the second most right, e.g., AUBES1884. The positional relationship of the conserved syntenies on the zebrafish genome sequence and within the catfish linkage group is indicated by thin lines linking the zebrafish chromosome and the catfish linkage group positions. The positions of markers within the linkage group are shown on the furthest right in centi-Morgans.

Mapping of microsatellites from contigs with hits to zebrafish chromosome 13 allowed identification of a highly conserved chromosome between catfish and zebrafish. As shown in Figure 6, of the 129 microsatellites from BES with high similarities to the zebrafish chromosome 13, 57 microsatellites from 43 contigs were mapped into a single linkage group, spanning approximately 90 centi-Morgans, suggesting the conservation of a large segment of this chromosome. However, the entire chromosome is not conserved. The 129 microsatellites were mapped to a total of 24 linkage groups, with seven of the 24 linkage groups containing 4-12 markers (see Additional file 2).

**Figure 7:**
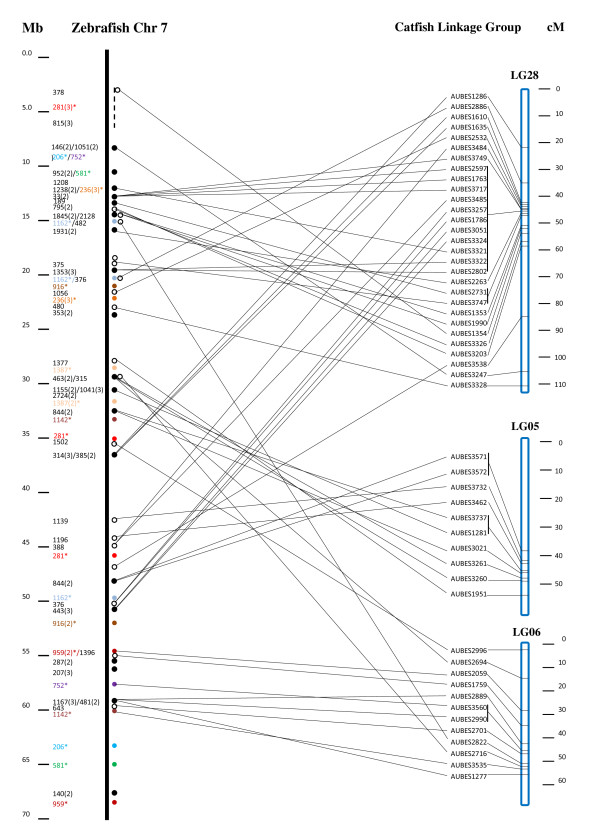
**Scaffolds of conserved syntenic regions between the catfish linkage groups and zebrafish chromosome 7**. Details of the methods used for the identification and presentation of the syntenic regions are the same as described under Figure 6.

Similarly but to a much lesser extent, microsatellites from BES with similarities to the zebrafish chromosome 7 were mapped to three major linkage groups (Figure 7). Once again, many smaller syntenic regions were mapped to various linkage groups, suggesting high levels of local conservation and low levels of chromosomal conservation. Nonetheless, the significant aspect of this is that scaffolds can be established by linking various contigs together through linkage mapping. This will allow integration of genetic linkage and physical maps once microsatellites are identified from most contigs of the physical map. Such scaffolds should guide genome sequence assembly in the future, and should also provide molecular length measurements of various polymorphic markers along the genome of catfish, providing guidance for the development of the SNP chip technology in catfish. Apparently, SNP chips constructed from evenly distributed SNPs provide the best coverage of the catfish genome when conducting the whole genome association studies.

Genetic linkage mapping of BAC end-anchored microsatellites provided a level of validation of the physical map. Discrepancies were found between the BAC assemblage and the linkage map. Of the 75 contigs with at least 2 markers, 54 contigs were mapped properly into the same linkage groups. However, 18 contigs were mapped into different linkage groups (see Additional file 2). Of these 18 contigs, 12 are large contigs with at least 40 BACs. Apparently, such discrepancy is indicative of mistakes in the BAC assemblage. Mapping additional BAC end-anchored microsatellites is under way to integrate the genetic linkage and physical maps, and to correct any additional mistakes in the assembly of the physical map [42].

## Conclusion

Some highly conserved chromosomes or chromosomal regions exist between catfish and zebrafish. High levels of local conservation were found, but a high level of chromosomal shuffling and rearrangements exists between catfish and zebrafish genomes. Comparative genome analysis using zebrafish genome sequence is highly useful for regional comparisons, but not so useful at the chromosomal levels. The significance of comparative genome analysis in catfish is that it will allow more cost-effective structural genomic analysis, but more importantly, orthologues established through comparative genome analysis should facilitate functional assignment of genes. Given that functional genomics is more difficult with non-model fish species, inference from orthologues should be one of the most efficient and reliable approaches for functional analysis of the catfish genome.

Overall, the evolutionary syntenic conservation appeared to be relatively low between the catfish genome and the genomes of the zebrafish. This indicates many chromosome breakage and rearrangements among the fish genomes occurred during evolution. These findings are consistent with our previous findings that high levels of conservation were found within small genomic regions, whereas high levels of large-scale genome reshuffling were evident when comparing the genomes of catfish and zebrafish [26,40]. These conclusions, however, are based on the assumption that the zebrafish genome assembly is correct. Apparently, due to the assembly mistakes in the zebrafish genome, some of the syntenic breaks may be due to the still poor assembly of the zebrafish genome. We also acknowledge that comparative genome analysis using a partial bank of sequences in catfish and a more complete databank in zebrafish could potentially lead to a bias. Caution should be exercised when establishing concrete syntenic relations. Such limitations themselves justify the need for whole genome sequencing in catfish.

## Methods

### BAC culture and BAC-end sequencing

The CHORI-212 Channel Catfish BAC library [47] was used for BAC-end sequencing. BAC culture and sequencing reactions were conducted using standard protocols, and as previously described [25,40]. Briefly, BAC clones were transferred from 384-well plates to 96-well culture blocks containing 1.5 ml of 2× YT medium with 12.5 μg/ml chloramphenicol and grown at 37°C overnight with shaking at 300 rpm. The blocks were centrifuged at 2000 × g for 10 min in an Eppendorf 5804R bench top centrifuge to collect bacteria. The culture supernatant was decanted and the blocks were inverted and tapped gently on paper towels to remove remaining liquid. BAC DNA was isolated using the Perfectprep™ BAC 96 kit (Eppendorf, Westbury, NY) according to the manufacturer's specifications. BAC DNA was collected in 96-well plates and stored at -20°C until usage.

Sequencing of channel catfish BAC ends was conducted using the BigDye^® ^Terminator v3.1 Cycle Sequencing Kit (Applied Biosystems, Foster City, CA), with modifications. Each sequencing reaction mix contained 2 μl of 5× sequencing buffer, 2 μl of primer (3 pmol/μl), 1.5 μl BigDye v3.1 dye terminator, and 4.5 μl of BAC DNA. BAC clones were sequenced from both ends using the primers T7 (5'-TAATACGACTCACTATAGGG-3') and SP6 (5'-ATTTAGGTGACACTATAG-3'). Cycle sequencing was carried out in 96-well plate format using PTC-200 thermal cyclers (MJ Research/Bio-Rad, Hercules, CA) under the following thermal profile: an initial denaturing at 95°C for 5 min, followed by 100 cycles of 95°C for 30 s, 53°C for 10 s, and 60°C for 4 min. Products were purified using ethanol/EDTA precipitation according to the BigDye protocol (Applied Biosystems), with the following modifications. After thermal cycling, 1 μl of 125 mM EDTA and 30 μl chilled (-80°C) 100% ethanol were added to each reaction. Plates were gently mixed and incubated at room temperature for 15 min. Plates were then centrifuged at 2,250 × g at 4°C for 30 min, followed by washing in 30 μl of 70% ethanol at 2,000 × g for 15 min. Ethanol was decanted and 8 μl Hi-Di™ formamide (Applied Biosystems) was added to each well to re-suspend DNA. Products were denatured at 95°C for 5 min and sequenced on a 3130*xl *genetic analyzer (Applied Biosystems).

### Sequence processing and analysis

The raw BES base calling were conducted by using Phred [48,49] with Q20 as a cut-off. Lucy program [50] was used to remove the vector sequences and short sequence less than 200 bp. Repeats were masked using REPEATMASKER [51] before BLAST analysis. In order to anchor the catfish BES to the zebrafish genome, TBLASTX searches of the repeat-masked BES were conducted against the ENSEMBLE zebrafish cDNA database (Assembly 7).

### Identification of conserved syntenies between catfish and zebrafish

In the absence of the whole genome sequence, we attempted to establish microsyntenies based on physical linkage of gene sequences. First, if the genes were identified from both sides of a single BAC clone (mate-paired BES), then they are physically linked with a distance of the BAC clone insert size. If the same two genes were found to be linked on the zebrafish genome in the same genome neighborhood, a microsynteny was established.

Initially, BES were analyzed by BLASTN (*E*-value ≤ -5) for the identification of mate-pairs with significant hits on both sides of the BAC insert. Mate-paired BES were analyzed by tBLASTX (*E*-value ≤ -5) for the identification of genes on both sides of the BAC insert. After identification, the two mate-paired genes in each BAC were used as queries to search for their chromosomal locations on the zebrafish genome. Conserved microsyntenies were declared when the mate-paired genes existed within a distance of 1.0 Mb within the zebrafish genome.

Syntenies were also established using genes within contiguous sequences (contigs) based on the catfish physical map [42]. Genes identified from BES were located along the catfish physical map. Genes identified within the same contig and located on the same zebrafish chromosome with comparable distances as estimated from the catfish BAC contig, an extended synteny was established.

### Construction of the catfish syntenic groups using linkagemaps

In order to assess the scope of microsyntenies, two zebrafish chromosomes, chromosome 7 and 13, were chosen for analysis. Chromosome 7 had the largest number of significant hits and chromosome 13 had a large number of contigs having two or more hits (suggestive of high level of syntenic conservation). Syntenies were established using microsatellite-based linkage mapping. A total of 548 microsatellite loci in the contigs which had significant BLASTN hits to the zebrafish chromosome 7 and 13 were tested using a hybrid catfish resource family, F_1_-2 (female blue-channel catfish hybrid) × Ch-6 (male channel catfish) with 64 progeny.

Microsatellites were identified and analyzed using Msatfinder [52] and Vector NTI 10.0 (Invitrogen, Carlsbad, CA) as we previously described [24]. Polymerase chain reaction (PCR) primers were designed using Msatfinder [52]. Mononucleotide repeats were manually excluded. PCR amplification was conducted as previously described [24]. Briefly, each microsatellite PCR reaction contained 1× PCR buffer, 2 mM MgCl_2_, 0.2 mM of each dNTP, 4 ng upper primer, 6 ng lower primer, 1 pmol labeled primer, and 0.25 U of JumpStart *Taq *polymerase (Sigma, St. Louis, MO), and 20 ng genomic DNA. PCR amplification was carried out using a touchdown program with the following thermal profile: an initial denaturation at 94°C for 3.5 min, followed by 94°C for 30 s, 57°C for 30 s, and 72°C for 30 s for 20 cycles as the first step, and at 94°C for 30 s, 53°C for 30 s, and 72°C for 30 s for 15 cycles as the second step. A final extension was performed at 72°C for 10 minutes. The PCR products were analyzed on 7% sequencing gels using the 4300 DNA Analyzer (LI-COR^® ^Biosciences, Lincoln, NE). After gel electrophoresis, loci were manually genotyped to determine allele segregation patterns and polymorphisms in the resource family.

The catfish linkage map was constructed using JoinMap version 4.0 software as we previously described [26] using the cross-pollinating (CP) coding scheme, which handles the data containing various genotype configurations with unknown linkage phases [53]. Linkage between markers was examined by estimating LOD scores for recombination rate, and map distances were calculated using the Kosambi mapping function. Significance of marker linkage was determined at a final LOD threshold of 3.0.

### Accession numbers

The BES generated from this study have been deposited in GenBank and were assigned accession numbers from [GenBank:FI857756] to [GenBank:FI900776] and the existing BES from [GenBank:DX083364] to [GenBank:DX103729] were also used for comparative genome analysis in this study.

## Authors' contributions

HL and YJ contributed equally and their contribution accounts for the major part of this study. SW and JA participated in data analysis and manuscript preparation. PN and BS assisted in developing microsatellite markers. PX assisted in culturing the BAC clone and extracted DNA. HK contributed major part in linkage mapping analysis. ZL supervised the entire study and prepared the manuscript. All authors read and approved the final manuscript.

## Supplementary Material

Additional file 1 **tBLASTX search results**. A portion of the tBLASTX search results used for the identification of microsyntenies. Query ID are sequence identification of catfish BAC end sequences used as queries; Subject ID is the identification of the sequence with significant tBLASTX hits at the E-values (E-values are coded in this Table. For instance, 0.29 means e^-29^, 0.06 means e^-6^). Chromosome number is provided in the column Chr, with starting position (Chr SS) and ending position (Chr SE) provided. The potential gene identities are detailed under Description. Click here for file

Additional file 2 **Primers used to map the BAC end associated microsatellites**. Ctg_ID is contig number on the catfish physical map; Contig size refers to the number of BAC clones within the contig; BAC_ID is BAC identification number; Upper and lower primer sequences, and the linkage groups the microsatellites were mapped are given in column E, F, and G, respectively. Click here for file
